# Increasing trend toward joint-preserving procedures for hip osteonecrosis in the United States from 2010 to 2019

**DOI:** 10.1186/s42836-023-00176-5

**Published:** 2023-05-01

**Authors:** Mitchell K. Ng, Andriy Kobryn, Ivan J. Golub, Nicolas S. Piuzzi, Che Hang Jason Wong, Lynne Jones, Michael A. Mont

**Affiliations:** 1 Department of Orthopaedic Surgery, Maimonides Medical Center, Brooklyn, NY 11219 USA; 2 Department of Orthopaedic Surgery, SUNY Downstate College of Medicine, Brooklyn, NY 11203 USA; 3 Department of Orthopaedic Surgery, Cleveland Clinic Foundation, Cleveland, OH 44195 USA; 4 Department of Orthopaedic Surgery, Johns Hopkins School of Medicine, Baltimore, MD 21205 USA; 5 Sinai Hospital of Baltimore, Rubin Institute for Advanced Orthopedics, Baltimore, MD 21215 USA

**Keywords:** Osteonecrosis, Total hip arthroplasty, Joint preservation, Surgical trends

## Abstract

**Introduction:**

The incidence of osteonecrosis of the femoral head is estimated at about 10 to 20,000 patients annually, and, when left untreated, 80% or more of cases progress to femoral head collapse. A series of joint-preserving procedures have been developed to prevent/delay the need for hip arthroplasty. The aim of this study was to provide a five-year update: (1) evaluating temporal trends of arthroplasty *vs*. joint-preservation techniques such as core decompression, bone grafting, osteotomies, and arthroscopy; (2) determining proportions of procedures in patients aged less than *vs*. over 50 years; and (3) quantifying rates of specific operative techniques.

**Methods:**

A total of 10,334 patients diagnosed with osteonecrosis of the femoral head and having received hip surgery were identified from a nationwide database between 1 January 2010 and 31 December 2019, by using the International Classification of Disease, the Ninth/Tenth revision (ICD-9/10) codes. The percentage of patients managed by each operative procedure was calculated annually. To identify trends, patients were grouped by age under/over 50 years and divided into a joint-preserving and a non-joint-preserving (arthroplasty) group. *Chi*-squared tests were performed to compare the total number of procedures per year.

**Results:**

Rates of arthroplasty far exceeded those for joint-preserving procedures. However, from 2015 to 2019, significantly more joint-preserving procedures were performed than in 2010 to 2014 (4.3% *vs*. 3.0%, *P* < 0.001). Significantly more joint-preserving procedures were performed in patients aged < 50 years relative to those ≥ 50 years (7.56% *vs.* 1.86%, *P* < 0.001). Overall, total hip arthroplasty was the most common procedure (9,814; 94.97%) relative to core decompression (331; 3.20%), hemiarthroplasty/resurfacing (102; 0.99%), bone grafting (48; 0.46%), and osteotomy (5; 0.05%).

**Conclusion:**

Management of patients who have osteonecrosis of the femoral head continues to be predominantly arthroplasty procedures, specifically, total hip arthroplasty. Our findings suggest a small, but significant trend toward increased joint-preserving procedures, especially in patients under 50 years. In particular, the proportion of patients receiving core decompression has increased significantly from 2015 to 2019 relative to prior years.

## Introduction

Osteonecrosis of the femoral head (ONFH), or hip osteonecrosis (ON), has an estimated incidence of 20,000 to 30,000 patients annually in the USA [1] and is thought to be the underlying diagnosis in about 10% of all total hip arthroplasties (THA) [2–4]. The pathophysiology of ONFH involves disruption of the vascular supply of subchondral bone leading to osteoblast death and articular surface collapse [1]. Although the specific etiologies are multifactorial and incompletely understood, they can be broadly divided into either direct causes (e.g., trauma, irradiation, dysbarism/Caisson disease, hematologic diseases, sickle cell disease) [5] or indirect causes (e.g. alcoholism, corticosteroid use, hypercoagulable states, systemic lupus erythematosus, protease inhibitors, viruses such as hepatitis or human immunodeficiency virus) [1, 4]. Patients between the ages of 35 to 50 are most susceptible and more than 80% of untreated cases will progress to femoral head collapse and arthritis [4]. Given the relatively young age of presentation and the known natural progression of hip ON [6, 7], there has been considerable interest in developing effective management with joint preservation.

While total hip arthroplasty remains the gold standard that provides good outcomes, with modern implants estimated to last 25 years in 58% of patients [8], when performed in patients who are young and relatively active, they may necessitate at least one revision in the future. Other non-joint-preserving procedures such as hemiarthroplasty or hip resurfacing likewise present similar revision concerns [9–11]. To this end, there has been growing interest in developing joint-preserving procedures, including core decompression, and bone grafting. Such joint-preserving options can be divided into more simple early-stage hip ON pre-collapse (e.g*.*, core decompression, bone grafting, percutaneous drilling with ancillary bone marrow aspirate concentrate) [1, 12–14] to more complex procedures (e.g. fibula bone graft, osteotomy, non-vascularized bone graft) [1, 12–16]. Although a wide range of joint-preserving procedures has been performed [1, 14, 17], to date, joint-preservation procedures remain a small minority of total procedures. To our knowledge, the most recent database study on ONFH was from 2009 to 2015, using the National Inpatient Sample, and found that joint-preserving procedures accounted for 4.9% and 1.5% for patients under and over 50 years of age respectively [18].

To this end, trends in management for ONFH should be updated to give healthcare providers the latest preferred forms of surgical treatment. The aim of this study was to (1) provide a five-year update and characterize overall annual trends in joint-preserving and non-joint-preserving procedures; (2) determine the proportion of joint-preserving *vs.* arthroplasty procedures for patients < 50 years relative to those ≥ 50 years of age; and (3) quantify the use of specific surgical procedures for these patients. Given the growing interest in joint preservation and its relative safety, we hypothesized that, over the past five years, there has been an increase in the proportion of joint-preserving procedures relative to arthroplasty techniques.

## Methods

### Database

This study retrospectively analyzed treatment trends using the American College of Surgeons (ACS) National Surgical Quality Improvement Program (NSQIP) database. One of the most commonly used databases in orthopaedic surgery research [19], this database prospectively collects data on patients undergoing major surgery from more than 700 participating hospitals in the United States via ACS-trained surgical clinical reviewers, who use validated, risk-adjusted methodologies with inter-rater reliability audits to guarantee validity. These quality assessment audits have a reported inter-rater disagreement rate of less than 1.8% [20]. Institutional review board (IRB) approval was not required for this study as the NSQIP database contains publicly available de-identified patient data.

### Patient selection

The NSQIP database was queried for all patients who were diagnosed with osteonecrosis of the femoral head between 1 January 2010 and 31 December 2019, using International Classification of Disease, the Ninth Revision (ICD-9) and Tenth Revision (ICD-10) diagnosis codes. Subsequently, ONFH patients were subdivided in terms of surgical management received during admission using Current Procedural Terminology (CPT) codes. Those classified under non-joint preserving procedures included total hip arthroplasty (27,130, 27,132, 27,134) and hemiarthroplasty/femoral head resurfacing (27,125). Joint-preserving procedures involved core decompression (27,299, 27,071, 26,922), bone grafting (20,955, 27,170), osteotomy (27,161, 27,165), and the unspecified (29,861, 29,862). Overall, 10,334 patients were identified who had a mean age of 55 years (range, 18 to 89 years), 4,441 women (43.0%) and 5,890 men (5,890; 57.0%). Furthermore, given that the diagnosis of ONFH is most common in patients aged 35 to 55 years of age, patients were separated according to age (≥ or < 50 years age), similar to previous studies) [18]. Of note, a significant increase in the total number of patients diagnosed with ONFH was observed from 2010 to 2019 (223 *vs.* 1,428 respectively) (*P* < 0.05). This growth may be attributed to the inclusion of more sites into the database. More specifically, the 2010 dataset contained data from 258 hospitals (363,431 total cases), whereas, the 2019 dataset contained data from 719 hospitals (1,076,411 total cases). To account for this variation, our analysis focused on the percentage/proportion of ONFH managed with various procedures within any given year rather than focusing on the absolute number of procedures performed.

### Statistical analyses

The total number and percentage of patients managed with joint-preserving and non-joint-preserving procedures for each study year was calculated and compared between years, for both patients under and over 50 years of age. *Chi*-squared tests were used to compare the total number of each procedure performed by year as well as to compare aggregate relative percentages. Trend analyses were conducted to determine whether the procedure type varied during the time of interest. All statistical analyses were conducted by using SPSS version 28.0 (IBM Corporation, Armonk, New York, NY, USA), with a *P*-value of 0.05 as the threshold for statistical significance.

## Results

### Trends in joint-preserving procedures relative to non-joint-preserving ones (Arthroplasty)

**Fig. 1:**
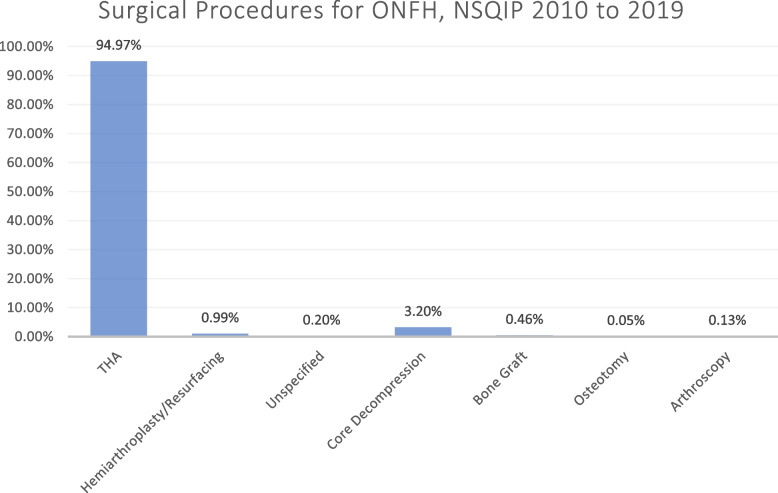
Surgical procedures for osteonecrosis of the femoral head (ONFH) based on the national surgical quality improvement program (NSQIP) data from 2010 to 2019

Overall, there has been a statistically significant increase in the proportion of joint-preserving procedures when comparing 2010–2014 to 2015–2019 (3.0% *vs.* 4.3%, *P* < 0.001) (Table 1). Specifically, there were significantly more core decompressions from 2010 to 2014 relative to 2015 to 2019 (2.3% *vs*. 3.6%, *P* < 0.001). Nevertheless, as a whole, from 2010 to 2019, only 397 procedures (3.8%) were joint-preserving, while 9,937 (96.2%) were non-join-preserving (Fig. 1).

**Table 1:** Trends in the type of procedures for all patients from 2009–2019

Year	Total hip arthroplasty *n* (%)	Hemiarthroplasty/resurfacing *n* (%)	Unspecified *n* (%)	Core decompression *n* (%)	Bone grafting *n* (%)	Osteotomy *n* (%)	Arthroscopy *n* (%)	Total *n* (%)
2010	207 (92.8)	3 (1.35)	2 (0.90)	4 (1.79)	3 (1.35)	1 (0.45)	3 (1.35)	223 (100)
2011	541 (95.8)	10 (1.77)	2 (0.35)	10 (1.77)	1 (0.18)	0 (0.00)	1 (0.18)	565 (100)
2012	640 (96.95)	11 (1.65)	2 (0.30)	12 (1.80)	2 (0.30)	0 (0.00)	0 (0.00)	667 (100)
2013	872 (95.51)	9 (0.99)	2 (0.22)	26 (2.85)	3 (0.33)	0 (0.00)	1 (0.11)	913 (100)
2014	1040 (95.50)	14 (1.29)	0 (0.00)	29 (2.66)	5 (0.46)	0 (0.00)	1 (0.09)	1089 (100)
**Total**	**3300 (95.40)**	**47 (1.36)**	**8 (0.23)**	**81 (2.34)**	**14 (0.40)**	**1 (0.03)**	**6 (0.17)**	**3457 (100)**
2015	1335 (94.82)	8 (0.57)	3 (0.21)	48 (3.41)	9 (0.64)	3 (0.21)	2 (0.14)	1408 (100)
2016	1256 (94.72)	6 (0.45)	1 (0.08)	49 (3.70)	11 (0.83)	1 (0.08)	2 (0.15)	1326 (100)
2017	1222 (92.86)	22 (1.67)	5 (0.38)	62 (4.71)	4 (0.30)	0 (0.00)	1 (0.08)	1316 (100)
2018	1304 (95.25)	10 (0.73)	2 (0.15)	49 (3.58)	4 (0.29)	0 (0.00)	0 (0.00)	1369 (100)
2019	1397 (95.82)	9 (0.62)	2 (0.14)	42 (2.88)	6 (0.41)	0 (0.00)	2 (0.14)	1458 (100)
**Total**	**6514 (94.72)**	**55 (0.80)**	**13 (0.19)**	**250 (3.64)**	**34 (0.49)**	**4 (0.06)**	**7 (0.10)**	**6877 (100)**
**Overall**	**9814 (94.97)**	**102 (0.99)**	**21 (0.20)**	**331 (3.20)**	**48 (0.46)**	**5 (0.05)**	**13 (0.13)**	**10,334**

### *Surgical procedural volumes by patient age* < *50 vs.* ≥ *50 years*

**Fig. 2:**
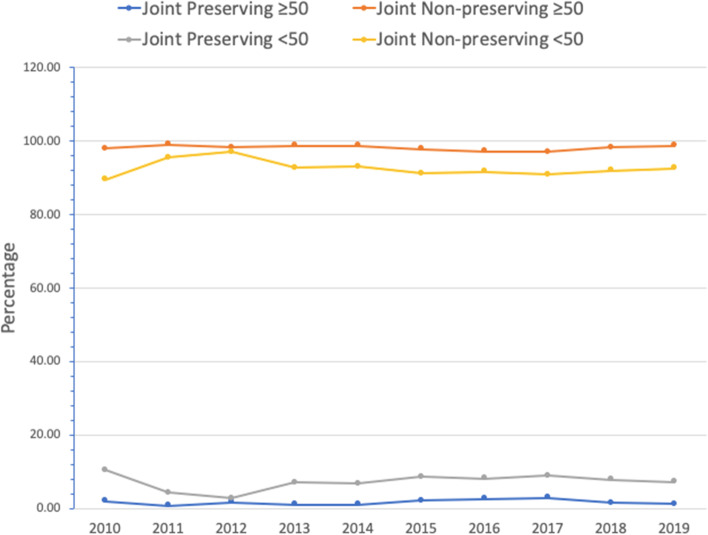
Trends in joint-preserving vs. non-joint-preserving procedures in patients aged above and below 50 years old from 2009–2019

There were significantly more joint-preserving operations in patients aged < 50 years (7.6% *vs*. 1.9%, *P* < 0.001) (Fig. 2). Among patients less than 50 years, there were 1,119/1192 non-preserving procedures (93.9%) and 73/1192 (6.1%) joint-preserving procedures from 2010 to 2014, and 2231/2432 non-joint-preserving procedures (91.7%) and 201/2432 (8.3%) joint-preserving procedures from 2015 to 2019 (Table 2). In contrast, among patients older than 50 years, there were 2206/2235 non-joint-preserving procedures (98.7%) and 29/2235 (1.3%) joint-preserving procedures from 2010 to 2014, and 4295/4389 (97.9%) and 94/4389 (2.1%) from 2015 to 2019 (Table 3).

**Table 2:** Trends in the type of procedures for patients younger than 50 from 2009–2019

Year	THA *n* (%)	Hemiarthroplasty/resurfacing *n* (%)	Unspecified *n* (%)	Core decompression *n* (%)	Bone grafting *n* (%)	Osteotomy *n* (%)	Arthroscopy *n* (%)	Total *n* (%)
2010	67 (88.16)	1 (1.32)	0 (0.00)	3 (3.95)	3 (3.95)	0 (0.00)	2 (2.63)	76 (100)
2011	190 (94.06)	2 (0.99)	1 (0.50)	8 (3.96)	0 (0.00)	0 (0.00)	1 (0.50)	202 (100)
2012	196 (95.61)	2 (0.98)	1 (0.49)	4 (1.95)	2 (0.98)	0 (0.00)	0 (0.00)	205 (100)
2013	290 (90.91)	5 (1.57)	1 (0.31)	20 (6.27)	2 (0.63)	0 (0.00)	1 (0.31)	319 (100)
2014	361 (92.56)	2 (0.51)	0 (0.00)	21 (5.38)	5 (1.28)	0 (0.00)	1 (0.26)	390 (100)
**Total**	**1104 (92.62)**	**12 (1.01)**	**3 (0.25)**	**56 (4.70)**	**12 (1.01)**	**0 (0.00)**	**5 (0.42)**	**1192 (100)**
2015	436 (90.83)	1 (0.21)	1 (0.21)	35 (7.29)	4 (0.83)	2 (0.42)	1 (0.21)	480 (100)
2016	442 (91.70)	0 (0.00)	0 (0.00)	29 (6.02)	8 (1.66)	1 (0.21)	2 (0.41)	482 (100)
2017	419 (89.91)	2 (0.43)	3 (0.64)	38 (8.15)	3 (0.64)	0 (0.00)	1 (0.21)	466 (100)
2018	448 (91.43)	3 (0.61)	0 (0.00)	35 (7.14)	4 (0.82)	0 (0.00)	0 (0.00)	490 (100)
2019	475 (92.41)	1 (0.19)	0 (0.00)	31 (6.03)	5 (0.97)	0 (0.00)	2 (0.39)	514 (100)
**Total**	**2220 (91.28)**	**7 (0.29)**	**4 (0.16)**	**168 (6.91)**	**24 (0.99)**	**3 (0.12)**	**6 (0.25)**	**2432 (100)**

**Table 3:** Trends in the type of procedures for patients aged 50 and above from 2009–2019

Year	THA *n* (%)	Hemiarthroplasty/resurfacing *n* (%)	Unspecified *n* (%)	Core decompression *n* (%)	Bone grafting *n* (%)	Osteotomy *n* (%)	Arthroscopy *n* (%)	Total *n* (%)
2010	139 (95.21)	2 (1.37)	2 (1.37)	1 (0.68)	0 (0.00)	1 (0.68)	1 (0.68)	146 (100)
2011	348 (96.94)	7 (1.95)	1 (0.28)	2 (0.56)	1 (0.28)	0 (0.00)	0 (0.00)	359 (100)
2012	436 (96.04)	9 (1.98)	1 (0.22)	8 (1.76)	0 (0.00)	0 (0.00)	0 (0.00)	454 (100)
2013	575 (98.12)	3 (0.51)	1 (0.17)	6 (1.02)	1 (0.17)	0 (0.00)	0 (0.00)	586 (100)
2014	671 (97.25)	11 (1.59)	0 (0.00)	8 (1.16)	0 (0.00)	0 (0.00)	0 (0.00)	690 (100)
**Total**	**2169 (97.05)**	**32 (1.43)**	**5 (0.22)**	**25 (1.12)**	**2 (0.09)**	**1 (0.04)**	**1 (0.04)**	**2235 (100)**
2015	895 (97.07)	5 (0.54)	2 (0.22)	13 (1.41)	5 (0.54)	1 (0.11)	1 (0.11)	922 (100)
2016	801 (96.51)	5 (0.60)	1 (0.12)	20 (2.41)	3 (0.36)	0 (0.00)	0 (0.00)	830 (100)
2017	795 (94.87)	16 (1.91)	2 (0.24)	24 (2.86)	1 (0.12)	0 (0.00)	0 (0.00)	838 (100)
2018	844 (97.46)	6 (0.69)	2 (0.23)	14 (1.62)	0 (0.00)	0 (0.00)	0 (0.00)	866 (100)
2019	911 (97.64)	8 (0.86)	2 (0.21)	11 (1.18)	1 (0.11)	0 (0.00)	0 (0.00)	933 (100)
**Total**	**4246 (96.74)**	**40 (0.91)**	**9 (0.21)**	**82 (1.87)**	**10 (0.23)**	**1 (0.02)**	**1 (0.02)**	**4389 (100)**

### Annual trends and incidences of specific surgical management techniques

During the study period, when evaluating the different types of operative procedures used, THA was by far the most commonly performed (9,814; 95.0%), followed by core decompression (331; 3.20%), hemiarthroplasty/resurfacing (102; 0.99%), bone grafting (48; 0.5%), the unspecified (21; 0.20%), and osteotomy (5; 0.05%) (Fig. 1). While the rates of THA remained largely constant from 2010 to 2014 relative to 2015 to 2019 (95.4% *vs.* 94.7%, *P* > 0.05), during these two time periods there was a decrease in proportions of hemiarthroplasty/resurfacing (1.4% *vs.* 0.8%, *P* < 0.001) and an increase in core decompression (2.3% *vs.* 3.6%, *P* < 0.001) (Table 1).

## Discussion

ONFH is believed to be involved in the underlying diagnosis for 10% of all THA performed in the USA [1, 4]. Although THA continues to be the most commonly used procedure for pain relief with good outcomes, factors that should be considered for joint preservation include the presence/absence of symptoms, degree/stage of ON, location/extent of bony involvement, patient's age, as well co-morbidities [3, 18]. Our study found that while rates of arthroplasty continue to be far higher than those of joint-preserving procedures, from 2015 to 2019 there were significantly more joint-preserving procedures relative to 2010 to 2014 (4.3% *vs*. 3.0%, *P* < 0.001). As expected, rates of joint-preserving procedures continue to be significantly higher in patients < 50 years relative to those ≥ 50 years (7.6% *vs*. 1.9%, *P* < 0.001). Overall, though THA remains the most common procedure, accounting for 94.9% of the procedures, the next standard procedure is core decompression, which accounts for 3.2% of all operations.

The results from this study are in agreement with the current literature. To our knowledge, the most recent study examining the trends in ONFH management by Sodhi et al. recruited 219,371 patients and found that only 4.93% of procedures were joint-preserving. Notably, they found a decreasing rate of joint-preserving procedures from 2009 to 2015, while our most recent data demonstrated an increase in joint-preserving procedures from 2015 to 2019. This appears to be largely due to a rise in core decompression procedures, whose rates increased when comparing 2010 to 2014 and 2015 to 2019 (2.3% *vs.* 3.6%, *P* < 0.001). Core decompression procedures saw increased interest and advancement, with modern techniques using small-diameter percutaneous drilling [12], use of ancillary growth/differentiation factors (e.g. autologous bone marrow, demineralized bone matrix, bone morphogenetic proteins), and adjunctive vascular grafting [13, 16]. A meta-analysis of 47 studies suggested that recent core decompression techniques have led to better clinical and radiographic outcomes relative to prior techniques [21]. Of note, while there have been promising results with these experimental techniques including autologous stem cell implantation [22], core decompression with/without augmentation still has high failure rates with known preoperative femoral head subchondral collapse (Ficat stage III/IV hips) and modified Kerboul necrotic angles over 250 degrees [23]. While it is promising that the rates of joint-preserving procedures have risen relative to prior years, additional research is required to more fully characterize the indications and contraindications for joint preservation over arthroplasty.

In accordance with previous literature, our study found that significantly more joint-preserving procedures were performed for patients aged < 50 years relative to those ≥ 50. Although THA provides an excellent and consistent improvement in pain relief and functional outcomes, they often necessitate at least one revision in younger, active patients due to issues such as aseptic acetabular/femoral component loosening, polyethylene wear, and infection [24]. Accordingly, it is promising to see significantly more joint-preserving procedures performed in patients < 50 years. A review by Pierce et al. discussing prior hip-preserving procedures found that previous history of core decompression, bone grafting, rotational osteotomy and resurfacing did not have a negative impact on postoperative outcomes following THA [25]. Given their low morbidity, shorter operative time, and lower overall cost, joint-preserving procedures should be considered by orthopedic surgeons when appropriate before arthroplasty in younger, active patients without contraindications [1, 18, 26].

Nevertheless, our data demonstrated that THA remains the procedure of choice in managing ONFH, accounting for 94.9% of patients who were diagnosed with ONFH over the past decade. Implant innovations such as ceramic-on-ceramic bearing surfaces, cementless prostheses with highly-porous fixation, and the use of ultra-high molecular weight polyethylene have further increased long-term survivorship of THA. Moreover, studies have shown that previous history of joint-preserving procedures is not associated with negative outcomes following THA [27]. Nevertheless, patients who have end-stage renal disease and/or a transplant, sickle cell disease, and Gaucher disease have been noted to be at higher revision risk after THA [28]. Our findings demonstrated that other procedures such as hemiarthroplasty, and resurfacing have largely fallen out of favor, potentially due to concerns about failure and advancements in THA and other joint-preserving techniques [29]. More complex procedures such as vascularized or non-vascularized bone graft trapdoor (e.g*.*, lightbulb and Phemister techniques) have also been explored with high efficacy at 4-year mean follow-up [26, 30]. However, for advanced-stage ONFH with subchondral collapse, few good long-term treatment options are available apart from arthroplasty. To this end, a better understanding of risk factors and early diagnosis/management of hip ON is likely the key.

Although this study analyzed a large sample size from the ACS-NSQIP which includes data on 300,000 plus cases from 686 hospitals annually, it is not without limitations [19]. As a database study, we identified patients and procedures using ICD-9/-10 and CPT codes which may contain coding errors. This being said, NSQIP is consistently maintained by surgical clinical reviewers who have averaged an inter-rater variability of 1.8% [31]. Of note, it was not possible to separate hemiarthroplasty from hip resurfacing, although given small absolute numbers this did not detract from our study. We were unable to identify the Ficat stage of osteonecrosis when patients presented for management, and were therefore unable to match the subsequent surgical option with disease stage [32]. The severity stratification of osteonecrosis along with baseline patient functional status could be investigated further by future prospective studies. In addition, our NSQIP study focused predominantly on identifying surgical trends and did not have the ability to distinguish between potential variable indications for surgery, and surgical approaches, or report long-term functional or patient-reported outcomes of these procedures. In addition, due to inavailability of CPT coding, we were unable to determine the rates or use of biological augmentation in core decompression. In spite of these limitations, as a whole, we believe that this study is well-powered to characterize surgical volume trends in the management of hip ON.

## Conclusion

The annual incidence of ONFH is estimated to stand at 10 to 20,000 annually and is believed to be the underlying diagnosis in 10% of all THAs performed in the USA. Due to its presentation in younger, active patients who are not ideal arthroplasty candidates due to the risk of at least one revision in the future, a series of joint-preserving procedures have been developed to delay/prevent progression. Our study found that the rate of joint-preserving procedures has risen relative to prior years, particularly in patients under the age of 50 years, and more specifically, the rates of core decompression have risen from 2015 to 2019 relative to previous years. This could potentially be explained by promising early results from modern techniques including small-diameter drilling with augmentation with bone marrow aspirates. Overall, our findings provide insight into current management trends of ONFH, of which THA remains dominant in nearly 94.9% of patients diagnosed with ONFH. Future work should be geared towards further characterizing appropriate surgical candidates for joint-preserving procedures and their subsequent outcomes.

## Data Availability

All data are publicly available to participating institutions. The ACS-NSQIP from which the data are derived have not been verified and are not responsible for the statistical validity of the data analysis or the conclusions derived by the authors.

## References

[1] Lespasio MJ, Sodhi N, Mont MA (2019). Osteonecrosis of the Hip: A Primer. Perm J.

[2] George G, Lane JM. Osteonecrosis of the Femoral Head. JAAOS Glob Res Rev 2022;6. 10.5435/JAAOSGLOBAL-D-21-00176. 10.5435/JAAOSGlobal-D-21-00176 PMC9076447 35511598

[3] Desforges JF, Mankin HJ. Nontraumatic Necrosis of Bone (Osteonecrosis). 2010;326:1473–9. 10.1056/NEJM199205283262206. 10.1056/NEJM199205283262206 1574093

[4] Treatment of nontraumatic hip osteonecrosis (avascular necrosis of the femoral head) in adults - UpToDate n.d. https://www.uptodate.com/contents/treatment-of-nontraumatic-hip-osteonecrosis-avascular-necrosis-of-the-femoral-head-in-adults (Accessed 17 Mar 2022).

[5] Mont MA, Hungerford DS (1995). Non-traumatic avascular necrosis of the femoral head. J Bone Joint Surg Am.

[6] Biz C, Berizzi A, Crimí A, Marcato C, Trovarelli G, Ruggieri P (2017). Management and treatment of femoral neck stress fractures in recreational runners: a report of four cases and review of the literature. Acta Biomed.

[7] Biz C, Tagliapietra J, Zonta F, Belluzzi E, Bragazzi NL, Ruggieri P (2020). Predictors of early failure of the cannulated screw system in patients, 65 years and older, with non-displaced femoral neck fractures. Aging Clin Exp Res.

[8] Evans JT, Evans JP, Walker RW, Blom AW, Whitehouse MR, Sayers A (2019). How long does a hip replacement last? A systematic review and meta-analysis of case series and national registry reports with more than 15 years of follow-up. Lancet (London, England).

[9] Cho YJ, Nam DC, Jung K (2014). Arthroplasty in Femoral Head Osteonecrosis. Hip Pelvis.

[10] Chalmers BP, Perry KI, Hanssen AD, Pagnano MW, Abdel MP (2017). Conversion of Hip Hemiarthroplasty to Total Hip Arthroplasty Utilizing a Dual-Mobility Construct Compared With Large Femoral Heads. J Arthroplasty.

[11] Grevitt MP, Spencer JD (1995). Avascular necrosis of the hip treated by hemiarthroplasty: Results in renal transplant recipients. J Arthroplasty.

[12] Mont MA, Ragland PS, Etienne G (2004). Core decompression of the femoral head for osteonecrosis using percutaneous multiple small-diameter drilling. Clin Orthop Relat Res.

[13] Calori GM, Mazza E, Colombo A, Mazzola S, Colombo M (2017). Core decompression and biotechnologies in the treatment of avascular necrosis of the femoral head. EFORT Open Rev.

[14] Pierce TP, Jauregui JJ, Elmallah RK, Lavernia CJ, Mont MA, Nace J. A current review of core decompression in the treatment of osteonecrosis of the femoral head. Curr Rev Musculoskelet Med 2015 83 2015;8:228–32. 10.1007/S12178-015-9280-0. 10.1007/s12178-015-9280-0 PMC4596206 26045085

[15] Seyler TM, Marker DR, Ulrich SD, Fatscher T, Mont MA (2008). Nonvascularized bone grafting defers joint arthroplasty in hip osteonecrosis. Clin Orthop Relat Res.

[16] Cao L, Guo C, Chen J, Chen Z, Yan Z (2017). Free Vascularized Fibular Grafting Improves Vascularity Compared With Core Decompression in Femoral Head Osteonecrosis: A Randomized Clinical Trial. Clin Orthop Relat Res.

[17] Mont MA, Professor D Sponseller AP, Professor S Hungerford AD, Einhorn TA, Mont MA, Einhorn TA, et al. The trapdoor procedure using autogenous cortical and cancellous bone grafts for osteonecrosis of the femoral head. 1998;80-B:56–62. 10.1302/0301-620X.80B1.0800056. 10.1302/0301-620x.80b1.7989 9460954

[18] Sodhi N, Acuna A, Etcheson J, Mohamed N, Davila I, Ehiorobo JO, et al. Management of osteonecrosis of the femoral head. 2020;102-B:122–8. 10.1302/0301-620X.102B7.BJJ-2019-1611.R1. 10.1302/0301-620X.102B7.BJJ-2019-1611.R1 32600203

[19] Ng MK, Vakharia RM, Bozic KJ, Callaghan JJ, Mont MA (2021). Clinical and Administrative Databases Used in Lower Extremity Arthroplasty Research. J Arthroplasty.

[20] Davis CL, Pierce JR, Henderson W, Spencer CD, Tyler C, Langberg R (2007). Assessment of the Reliability of Data Collected for the Department of Veterans Affairs National Surgical Quality Improvement Program. J Am Coll Surg.

[21] Marker DR, Seyler TM, Ulrich SD, Srivastava S, Mont MA (2008). Do modern techniques improve core decompression outcomes for hip osteonecrosis?. Clin Orthop Relat Res.

[22] Talathi NS, Kamath AF (2018). Autologous stem cell implantation with core decompression for avascular necrosis of the femoral head. J Clin Orthop Trauma.

[23] Boontanapibul K, Huddleston JI, Amanatullah DF, Maloney WJ, Goodman SB (2021). Modified Kerboul Angle Predicts Outcome of Core Decompression With or Without Additional Cell Therapy. J Arthroplasty.

[24] Betsch M, Tingart M, Driessen A, Quack V, Rath B (2018). Total hip replacement in avascular femoral head necrosis. Orthopade.

[25] Pierce TP, Elmallah RK, Jauregui JJ, Verna DF, Mont MA (2015). Outcomes of total hip arthroplasty in patients with osteonecrosis of the femoral head—a current review. Curr Rev Musculoskelet Med.

[26] Marker DR, Seyler TM, McGrath MS, Delanois RE, Ulrich SD, Mont MA (2008). Treatment of early stage osteonecrosis of the femoral head. J Bone Jt Surg - Ser A.

[27] Issa K, Pivec R, Kapadia BH, Banerjee S, Mont MA. Osteonecrosis of the femoral head: the total hip replacement solution. Bone Joint J 2013;95-B:46–50. 10.1302/0301-620X.95B11.32644/ASSET/IMAGES/LARGE/32644-GALLEYFIG4.JPEG. 10.1302/0301-620X.95B11.32644 24187351

[28] Johannson HR, Zywiel MG, Marker DR, Jones LC, McGrath MS, Mont MA (2011). Osteonecrosis is not a predictor of poor outcomes in primary total hip arthroplasty: a systematic literature review. Int Orthop.

[29] Sawadogo M, Kafando H, Ouedraogo S, Korsaga AS, Ouedraogo S, Tinto S (2018). Is Head and Neck Resection of the Femur (Girdlestone’s Procedure) Still Relevant? Indications and Results About 24 Cases. Open Orthop J.

[30] Mont MA, Etienne G, Ragland PS (2003). Outcome of Nonvascularized Bone Grafting for Osteonecrosis of the Femoral Head. Clin Orthop Relat Res.

[31] Khuri SF, Daley J, Henderson WG (2002). The comparative assessment and improvement of quality of surgical care in the Department of Veterans Affairs. Arch Surg.

[32] Sultan AA, Mohamed N, Samuel LT, Chughtai M, Sodhi N, Krebs VE (2019). Classification systems of hip osteonecrosis: an updated review. Int Orthop.

